# Syndromic forms of congenital hyperinsulinism

**DOI:** 10.3389/fendo.2023.1013874

**Published:** 2023-03-30

**Authors:** Martin Zenker, Klaus Mohnike, Katja Palm

**Affiliations:** ^1^ Institute of Human Genetics, University Hospital, Otto-von-Guericke University Magdeburg, Magdeburg, Germany; ^2^ Department of Pediatrics, University Hospital, Otto-von-Guericke University Magdeburg, Magdeburg, Germany

**Keywords:** congenital hyperinsulinism, hyperinsulinemic hypoglycemia, Beckwith-Wiedemann syndrome, Sotos syndrome, Costello syndrome, Kabuki syndrome, chromosomal disorders

## Abstract

Congenital hyperinsulinism (CHI), also called hyperinsulinemic hypoglycemia (HH), is a very heterogeneous condition and represents the most common cause of severe and persistent hypoglycemia in infancy and childhood. The majority of cases in which a genetic cause can be identified have monogenic defects affecting pancreatic β-cells and their glucose-sensing system that regulates insulin secretion. However, CHI/HH has also been observed in a variety of syndromic disorders. The major categories of syndromes that have been found to be associated with CHI include overgrowth syndromes (e.g. Beckwith-Wiedemann and Sotos syndromes), chromosomal and monogenic developmental syndromes with postnatal growth failure (e.g. Turner, Kabuki, and Costello syndromes), congenital disorders of glycosylation, and syndromic channelopathies (e.g. Timothy syndrome). This article reviews syndromic conditions that have been asserted by the literature to be associated with CHI. We assess the evidence of the association, as well as the prevalence of CHI, its possible pathophysiology and its natural course in the respective conditions. In many of the CHI-associated syndromic conditions, the mechanism of dysregulation of glucose-sensing and insulin secretion is not completely understood and not directly related to known CHI genes. Moreover, in most of those syndromes the association seems to be inconsistent and the metabolic disturbance is transient. However, since neonatal hypoglycemia is an early sign of possible compromise in the newborn, which requires immediate diagnostic efforts and intervention, this symptom may be the first to bring a patient to medical attention. As a consequence, HH in a newborn or infant with associated congenital anomalies or additional medical issues remains a differential diagnostic challenge and may require a broad genetic workup.

## 1 Introduction

Congenital hyperinsulinism (CHI), also termed congenital hyperinsulinemic hypoglycemia (HH), is a disorder of glucose homeostasis due to dysregulated insulin secretion in the newborn or young infant and represents the most common cause of severe and persistent hypoglycemia in infancy and childhood (1). The presenting symptom of CHI is persistent hypoglycemia typically manifesting shortly after birth with inappropriate insulin levels or indirect signs of inappropriate insulin action such as low plasma concentrations of ketone bodies and free fatty acids, as well as a positive glycemic response to glucagon at the time of hypoglycemia (2). Glucose utilization is increased and leads to high glucose infusion rates required to maintain euglycemia. Affected newborns typically have a high birth weight for gestational age, as fetal insulin secretion promotes *in utero* growth *via* insulin-like growth factor 1 receptor-mediated signaling (3).

CHI is clinically and pathogenetically very heterogeneous. It may be transient and permanent and has a strong genetic contribution. The majority of cases in which a genetic cause can be identified have monogenic defects affecting pancreatic β-cells and their glucose-sensing system that regulates insulin secretion. Such cases typically have a non-syndromic clinical constellation, i.e., no other primary organ manifestations besides the metabolic-endocrine abnormality. Alterations in the genes ABCC8 and KCNJ11 encoding components of the voltage-dependent K_ATP_ channel predominate. They can lead to either diffuse forms of pancreatic involvement due to recessive and dominant mutations, or to focal CHI caused by a unique mechanism involving a heterozygous loss-of-function variant on the paternal allele plus loss of heterozygosity of the 11p15.5 region encompassing the ABCC8/KCNJ11 locus due to a second somatic event (mostly a paternal uniparental disomy of 11p, patUPD11p) in the focal lesion (4). The cause of CHI still remains unknown in up to 50% of patients, which may be due to additional hitherto unidentified genetic loci for monogenic types of CHI (5), as well as the contribution of complex/multifactorial etiologies. In a small fraction of cases, CHI has found to be associated with syndromic or multisystemic diseases (reviewed by 5-8). Few of these syndromic disorders have been associated with genes playing a known direct role in the regulation of glucose metabolism, while for the majority of them the molecular mechanism leading to inappropriately elevated insulin secretion is still unclear. This article reviews syndromic disorders that have been asserted by the literature to be associated with CHI/HH. An overview of disease categories and individual conditions is presented in **Table 1**. We assess the evidence of the association, as well as the frequency of CHI, its epigenetic and genomic basis, possible pathophysiology and natural course in the respective conditions.

**Table 1:** Summary on syndromic diseases having suspected of proven association with CHI/HH.

Disease category	Disease	Inheri-tance	Locus	Involved gene(s)	Key features	Prevalence of neonatal hypoglycemia	CHI/HH association	Treatment and response	Course and outcome
**Overgrowth syndromes**	Beckwith-Wiedemann syndrome	Sporadic, AD	11p15.5	Genes of the 11p15.5 DMR; KCNQ1	Hemihyperplasia, macroglossia, omphalocele, visceromegaly	~50%	***patUPD11p***: >30%; few cases with additional K_ATP_ mutation	Often (70%) unresponsive to DXZ; almost half of reported cases received pancreatectomy	Most cases with persistent HI
							***Epimutations***: <5%	DXZ-reponsive in cases requiring treatment	Most cases with transient HI
	Sotos syndrome	AD	5q35.3	NSD1	Macrosomia, macrocephaly, DD/ID, dysmorphic features	<15%	Very rare, 25 cases reported	DXZ-reponsive; no case of pancreatectomy reported	Transient HI; mostly resolving within the first months of life
	Simpson-Golabi-Behmel syndrome	XL	Xq26.2	GPC3	Macrosomia, DD/ID, dysmorphic features	Increased, not specified	Not documented	NA	NA
	Weaver syndrome	AD	2q27.1	EZH2	Macrosomia, DD/ID, dysmorphic features	Increased, not specified	Not documented	NA	NA
	Perlman syndrome	AR	2q27.1	DIS3L2	Neonatal macrosomia, MCA, nephroblastomatosis, dysmorphic features	Several cases reported	Not documented	NA	NA
	PI3K-AKT pathway disorders	Sporadic, AD	19q13.2, 3q26.3, 1q43-q44, 14q32.33, 12p13.32	AKT2, PIK3CA, AKT3, AKT1, CCND2	Regional overgrowth, vascular malformations, epidermal nevi	Increased frequency of hypoglycemia in infancy and thereafter, not specified	Hypoinsulinemic hypoglycemia (CHI phenocopy), predominantly with AKT2	Unresponsive to DXZ and octreotide; may be managed with regular carbohydrate feeds; response to sirolimus reported	Variable course; spontaneous remission reported
**Monogenic develop-menttal disorders**	Kabuki syndrome	AD	12q13.12, Xp11.3	KMT2D, KDM6A	DD/ID, dysmorphic features, CHD, MCA, postnatal growth defect	<7%	<2%, predominantly with KDM6A	Usually DXZ-responsive; few cases of pancreatectomy reported	Variable course; may require treatment for several years
	Costello syndrome	AD	11p15.5	HRAS	Dysmorphic features, CHD, DD/ID, postnatal growth defect	~44%	Rare	Usually DXZ-responsive; one case of pancreatectomy reported	Persistence up to 6 months reported
	Rubinstein-Taybi syndrome	AD	16p13.3, 22q13.2	CREBBP, EP300	DD/ID, dysmorphic features, MCA, growth defect	Increased, not specified	Very rare	Limited data; more than half of patients responded to DXZ	Variable course; may require treatment for several years
	Coffin-Siris syndrome	AD	Multiple (12)	Multiple (12)	DD/ID, dysmorphic features, MCA	Anecdotal reports	Few cases reported	NA	NA
	CHARGE syndrome	AD	8q12.2	CHD7	MCA, CHD, dysmorphic features, DD/ID	Anecdotal reports	Only 2 cases reported	NA	NA
	FOXA2-CHI	AD	20p11.21	FOXA2	Hypopituitarism, MCA, DD/ID, dysmorphic features	Few cases reported	Few cases reported	Limited data; (partial) response to DXZ	Mostly transient; may be followed by impaired glucose tolerance and diabetes
	MEHMO syndrome	XL	Xp22.11	EIF2S3	DD/ID, hypopituitarism, epilepsy, obesity	Several cases reported	Few cases reported	Limited data; DXZ may improve glycemic response	Hyperinsulinemic hypoglycemia and postprandial hyperglycemia, diabetes may emerge
	Congenital central hypoventilation syndrome	AD	4p13	PHOX2B	Central hypoventilation, Hirschsprung disease	Episodic hypoglycemia reported in several cases	Several cases reported	Limited data; response to DXZ reported	Patients may exhibit postprandial hyperglycemia followed by (asymptomatic) hypoglycaemia
	CHI with renal tubular and hepatic dysfunction	AD	20q13.12	HNF4A (p.R76W)	Renal Fanconi syndrome, hepatic dysfunction	Probably >50%	Several cases reported	Limited data; mostly responsive to DXZ	Mostly transient; may be followed by impaired glucose tolerance and diabetes
	Schaaf-Yang syndrome	AD	15q11.2	MAGEL2	DD/ID, contractures, dysmorphic features	Unknown	Two unrelated observations	Limited data; variable response to DXZ	Few data; DZX treatment up to age 6 reported
**Chromoso-mal (contiguous gene) syndromes**	9p deletion syndrome	Sporadic, AD	9p24.1-24.2	SMARCA2, RFX3 (?)	DD/ID, dysmorphic features, MCA	Unknown	Several cases reported	DZX-responsive	Variable course; may require treatment for several years
	Turner syndrome	Sporadic	Xp	KDM6A (?)	Growth defect, hypogonadism, dysmorphic features	Increased, not specified	Several cases reported	Variable response to DXZ; 4 cases with pancreatectomy	Variable course; may require treatment for several years
	Usher-CHI syndrome	AR	11p15.1	ABCC8	Sensorineural deafness, retinitis pigmentosa	100%	100%	DXZ-resistant; most cases received pancreatectomy	Persistent HI
	Trisomy 13	Sporadic	13	Unknown	MCA, dysmorphic features, severe DD/ID	Unknown	Few cases reported	Variable response to DZX	Outcome dictated by underlying disease
	16p11.2 microdeletion	AD	16p11.2	Unknown	Non-specific DD/ID	Unknown	Very rare; few cases reported	Limited data; may respond to DZX	Limited data
	Trisomy 21	Sporadic	21	Unknown	Dysmorphic features, MCA, DD/ID	Unknown	Increased frequency in cohorts tested for CHI	Limited data; mostly DZX-responsive; 1 case of pancreatectomy	Mostly transient; remission within the first year
**Channelo-pathies**	Timothy syndrome	AD	12p13.33	CACNA1C	CHD, long-QT syndrome, syndactyly, DD/ID, dysmorphic features	Intermittent hypoglycemia in ~40%	Not documented	Limited data; may respond to DZX	Recurrent episodes of hypoglycemia
	PASNA	AD	3p21.1	CACNA1D	Aldosteronism, epilepsy, hypotonia, DD/ID	Increased, not specified	Two cases reported	Limited data; may respond to DZX or Ca2+ channel blockers	Limited data; DZX treatment up to age 4 reported
	Long-QT syndrome	AD	11p15.5, 7q36.1	KCNQ1, KCNH2	QTc prolongation, cardiac arrhythmia	Intermittent hypoglycemia reported	Hyperinsulinemia after glucose challenge	Limited data; usually no medical treatment required	Postprandial hyperinsulinemia and reactive hypoglycemia
**Metabolic disorders**	CDG syndrome	AR	16p13.2, 15q24.2, 3q27.1, 1p31.3	PMM2, MPI, ALG3, PGM1	DD/ID, brain and ocular anomalies, hepatopathy, enteropathy, coagulopathy, cystic kidneys	<10-89%, depending on CDG type; PMM2-HI: separate clinical and genetic subtype	Several cases reported	Usually DZX-responsive; may also improve with oral mannose; 1 case of pancreatectomy;	Variable course; may require prolonged DZX treatment
	Tyrosinemia	AR	15q25.1	FAH	Hepatic dysfunction, renal tubular dysfunction, DD/ID	Occasional	Few cases reported	DZX-responsive; specific treatment with NTBC	Mostly transient; dependent on metabolic control
	Adenosine kinase deficiency	AR	10q22.2	ADK	DD/ID, epilepsy, hypermethioninemia, dysmorphic features, CHD	Increased, not specified	Few cases reported	DZX-responsive; specific treatment with methionine restriction	Mostly transient; dependent on metabolic control

CHD, congenital heart defect; CHI, congenital hyperinsulinism; DD/ID, developmental delay/intellectual disability; DMR, differentially methylated region; DZX, diazoxide; HH, hyperinsulinemic hypoglycemia; HI, hyperinsulinism; MCA, multiple congenital anomalies; NA, no data available.

## 2 Methods

We conducted a systematic PubMed search for original studies and case reports, to identify original data on published syndromic cases of CHI regarding clinical presentation, genetic basis, pathophysiology, diagnosis, and management. General searches were performed using the search term combinations: (“congenital hyperinsulinism”) AND (syndrome OR syndromic), as well as: (“congenital hyperinsulinism”) AND (Review[Publication Type]), yielding 177 and 183 results, respectively. A separate search was performed for each CHI-associated entity identified through the retrieved original articles and reviews. The search term combination used were: “congenital hyperinsulinism” OR “neonatal hyperinsulinism” OR (hypoglycemia AND insulin) OR hyperinsulinemia OR hyperinsulinemic together with disease-specific terms. For each entity the search terms included disease name and name of disease-causing genes; as an example, the search regarding Sotos syndrome was: ((congenital hyperinsulinism) OR (neonatal hyperinsulinism) OR (hypoglycemia AND insulin) OR (hyperinsulinemia) OR (hyperinsulinemic)) AND ((“Sotos syndrome”) OR (NSD1)). The reference lists of the identified papers were also used to identify further papers of interest. The final reference list was selected on the basis of relevance to this study. A total of 1144 articles were retrieved by the various searches (excluding the search regarding PI3K-AKT pathway disorders that are not associated with hyperinsulinemia); 176 were selected for data collection. Duplicates of reported cases were excluded, if prior publication in another selected reference was stated by authors. From 27 references where full-text articles were not accessible, available data were extracted from abstracts. The full lists of references and summaries of collected data are presented in **Supplementary Table S1**.

## 3 Results

### 3.1 Beckwith-Wiedemann syndrome

**Figure 1:**
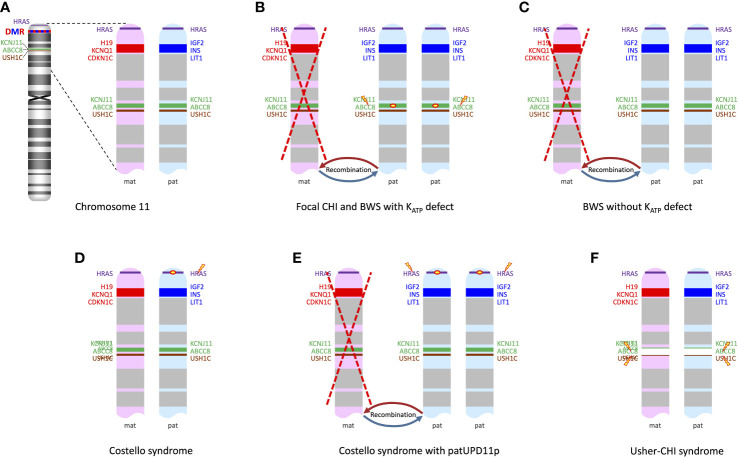
**(A)** ideogram of chromosome 11 displaying the loci of genes of interest in the context of syndromic CHI and the differentially methylated region (DMR) in the 11p15 region; enlarged view of 11p13-11p15 shows genes expressed from the maternal chromosome (in red) and those expressed from the paternal chromosome (in blue). **(B)** patUPD11p with a pathogenic variant in ABCC8 or KCNJ11 on the paternal allele leads to a biallelic defect of ABCC8 or KCNJ11 and at the same time to overexpression of paternally imprinted genes IGF2, INS and LIT1; this mechanism is shared by focal CHI and rare cases of BWS with an ABCC8 or KCNJ11 mutation on the paternal allele. **(C)** BWS due to patUPD11p without an ABCC8 or KCNJ11 mutation just has the overexpression of paternally imprinted genes, while expression of the maternally imprinted genes such as KCNQ1 is decreased or lacking. **(D)** In Costello syndrome, the pathogenic HRAS mutation usually resides on the paternally inherited chromosome; other genes in the region are unaffected. **(E)** Somatic patUPD11p in Costello syndrome leads to the overexpression of paternally imprinted genes/lack of expression of maternally imprinted genes and also to a duplication of the mutated HRAS, which may contribute to dysregulation of proliferation/differentiation in affected cells. **(F)** Usher-CHI syndrome results from a homozygous contiguous gene deletion encompassing parts of ABCC8 and the USH1C genes.

The characteristic clinical features of BWS (OMIM #130650) include fetal/neonatal macrosomia, macroglossia that is often asymmetric, hemihyperplasia, omphalocele or umbilical hernia, visceromegaly involving liver, spleen, kidneys, adrenal glands, and/or pancreas, as well as a predisposition to embryonal tumors (6, 7). In typical cases the diagnosis can quite easily be suspected clinically, based on the combination of characteristic symptoms as mentioned above and other minor anomalies such as ear lobe creases and/or posterior helical ear pits, facial anomalies, nevus flammeus and others. However, BWS represents a clinical spectrum, and some affected newborns may only have few or even singular suggestive clinical findings (7). Most cases are sporadic, but a positive family history is present in about 15% of cases (6). The reported prevalence of BWS is ~1:10,000 live births (8). BWS is caused by epigenetic or genomic alterations leading to abnormal methylation at a distinct differentially methylated region in 11p15.5 (BWS critical region, **Figure 1A**), namely (i) loss of methylation of IC2 (imprinting center 2) on the maternal chromosome (~50%), (ii) gain of methylation of IC1 on the maternal chromosome (~5%), paternal uniparental disomy of 11p15.5 (patUPD11p; ~20%), or a heterozygous pathogenic variant on the maternal CDKN1C allele (~5%). Other genomic variants involving the chromosome 11p15.5 region including (micro)duplications, (micro)deletions, inversions or translocations account for a small fraction of cases (~1%). BWS-associated epigenetic and genomic changes may be mosaic due to postzygotic occurrence of the underlying (epi)mutation (6, 7). Mosaicism has to be considered particularly in oligosymptomatic cases and its demonstration may be challenging, requiring molecular studies in additional tissues (e.g., skin biopsy from the side of hyperplasia). Mosaicism is particularly frequent in cases with patUPD11p, and a severe BWS phenotype is associated with high levels of somatic mosaicism for this anomaly (9). Familial occurrence of BWS is mostly associated with pathogenic variants in CDKN1C.

The incidence of neonatal hypoglycemia in BWS has been reported to be approximately 50% (10, 11). However, in most cases of BWS hypoglycemia is mild and transient. Notably, the onset of hypoglycemia can occasionally be delayed for several days, or even months (6). In BWS children with mild and transient hypoglycemia, the metabolic disturbance may resemble the one seen in newborns with fetal macrosomia due to maternal diabetes. However, in a minority of cases with BWS, hypoglycemia is more severe and persistent, showing the characteristics of CHI. Some patients are responsive to diazoxide (12) and some may even require pancreatic surgery (12–14). The overall prevalence of clinically significant and persistent CHI in BWS has not been determined, but is probably less than 10% (11). However, hyperinsulinism is one of the 6 cardinal features used in the clinical scoring system for the diagnosis of BWS (7).

The pathophysiology of CHI in BWS is not fully understood, but there is strong evidence for significant genotype-phenotype correlations. In a retrospective study on 28 children with CHI and a wide range of BWS-associated features ranging from classical BWS to oligosymptomatic cases with only mild hemihypertrophy, mosaic paternal uniparental isodisomy for chromosome 11p (patUPD11p) was found in 26 out of 28 cases (93%), while only two cases with CHI and BWS were associated with hypomethylation at IC2; these two had only mild CHI (14). This indicates that patUPD11p, which accounts for approximately 20% of cases with BWS in general, is the genotype that is specifically associated with a manifestation of CHI in BWS. This particular genotype-association is supported by other reports (13, 15–18) (**Supplementary Table S1**).

Four of the 28 cases reported by Kalish et al. (14) and a couple of other reported cases (15, 17) have been found to carry a paternally inherited K_ATP_ variant in addition to their patUPD11p, thus explaining the CHI phenotype on the same mechanistic basis as the established dual hit mechanism for focal CHI (**Figure 1B**). Not surprisingly, these patients had a much more severe course of HH than patients with patUPD11p alone (14). Pancreatic histology showed areas of irregular proliferation of endocrine tissue forming coalescing nodules and trabeculae with a more widespread pattern compared to typical focal pancreatic lesions (14, 15), a pattern that probably reflects the distribution of mosaicism for the patUPD11p in the pancreas. Therefore, the spectrum of disorders caused by the combination of a paternally inherited K_ATP_ channel mutation and a secondary somatic patUPD11p can be regarded as a continuum where the timing and distribution of the second event is critical: BWS symptoms represent the clinical correlate of an early embryonic occurrence leading to systemic involvement with patUPD11p, while late emergence of patUPD11p that is restricted to the pancreas causes non-syndromic focal CHI (19).

However, the majority of cases with CHI and BWS due to patUPD11p reported by Kalish et al. were not associated with a K_ATP_ variant, suggesting that the UPD11p *per se* can also initiate CHI with a K_ATP_-independent mechanism (14) (**Figure 1C**). The authors proposed a combined mechanism of expanded β-cell mass due to the pro-proliferative effect of the paternal imprint pattern at the 11p15 region together with functional abnormalities in β-cell insulin secretion possibly caused by the lack of the maternally expressed KCNQ1 voltage-gated potassium channel that is assumed to be involved in the regulation of potassium flux in pancreatic β-cells (14). Consistent with the assumption that patUPD11p in the pancreas can *per se* cause CHI, Flanagan et al. reported two cases of CHI with segmental patUPD11p in DNA extracted from pancreatic tissue and no K_ATP_ channel mutation (20). patUPD11p was not found in DNA from leukocytes and buccal cells and the patients were lacking clinical signs of BWS.

It has been reported that most of the patUPD11p patients without a paternally inherited K_ATP_ channel mutation do not require treatment beyond 2 years of age (12, 14). This might be related to the shift of KCNQ1 expression from monoallelic fetal to biallelic adult expression (21). Notably, in the subgroup of cases with patUPD11p retrieved by our literature search, persistent HI is more common, resistance to diazoxide prevails, and 17 of 48 cases with patUPD11p alone, as well as all cases with an additional K_ATP_ mutation received partial or subtotal pancreatectomies (**Supplementary Table S1**). A reporting bias towards more severely affected individuals and unidentified duplicates cannot be excluded.

**Mosaic genome-wide paternal UPD** is a very rare disorder with a predominant phenotype of patUPD11 (BWS-like) and a high risk of tumor development, which may by expanded by features of other patUPD-related disorders (22). CHI is a frequent complication (22, 23) and follows the same mechanism as in patUPD11p-related CHI discussed above. Similarly, a high rate of unresponsiveness to diazoxide and need for surgery was reported in published cases (**Supplementary Table S1**). **Multilocus imprinting disturbance** (MLID) is a primary epigenetic disorder where aberrant imprinting marks (most commonly loss of methylation) occur at multiple differentially methylated regions of the genome. It may be caused by defects in the subcortical maternal complex (SCMC) which is required for the proper oocyte maturation and early embryonic development (24). BWS-like features may be accompanied by symptoms of other imprinting disorders. Similar to BWS caused by epimutations, hypoglycemia may occur, but overt CHI is probably very rare.

### 3.2 Sotos syndrome

Sotos syndrome (OMIM #117550) is characterized by overgrowth (macrosomia and/or macrocephaly) with advanced bone maturation, developmental/neuropsychological deficits, and a distinctive facial appearance including a prominent forehead, dolichocephaly, sparse frontotemporal hair, downslanting palpebral fissures, malar flushing, and a long face. Affected individuals may also display cardiac or renal anomalies, musculoskeletal abnormalities, seizures, and a variety of other abnormalities. Sotos syndrome is estimated to occur in 1:14,000 live births (25). Sotos syndrome can be caused by a heterozygous pathogenic variant in NSD1 or a deletion encompassing NSD1. In the vast majority of cases the causative variant is *de novo* (25).

Neonatal hypoglycemia is a known feature of Sotos syndrome but it has been observed in less than 15% of affected individuals (25). However, several well-documented cases with Sotos syndrome and CHI have been reported (26–30). In the first reported cases, NSD1 microdeletions predominated, leading to the speculation that additional genes in the deleted 5q35 region might be critical for the metabolic abnormality (26, 27). However, subsequent reports on several patients with Sotos syndrome and CHI who carried intragenic NSD1 mutations rather suggest that the defect in NSD1 itself is sufficient to cause CHI (28–30).

The precise mechanism of dysregulated insulin secretion in Sotos syndrome is unknown. Notably, the gene product of NSD1 is a histone methyltransferase that is known to be involved in the regulation of chromatin and gene expression, and circumstantial evidence exists that NSD1 may thereby also regulate islet cell insulin expression (30).

In the majority of cases of Sotos syndrome CHI was transient, but Grand et al. also reported three patients with CHI persisting up to the age of 4 years (30). Responsiveness to diazoxide treatment was commonly observed (30), and no case of pancreatectomy has been reported (**Supplementary Table S1**).

### 3.3 Other overgrowth syndromes

For **Malan syndrome** (also known as Sotos syndrome 2, OMIM #614753), no cases of CHI have been reported to date (31).

**Simpson-Golabi-Behmel syndrome** (SGBS; OMIM #312870**)** is characterized by pre- and postnatal macrosomia, distinctive craniofacial features, intellectual disability with or without structural brain anomalies, and variable other anomalies. It is caused by pathogenic variants in GPC3 and inherited in an X-linked manner with possible disease manifestation also in females. Neonatal hypoglycemia was mentioned as a possible complication “as in other overgrowth syndromes” in a review on SGBS (32), but its frequency is unknown and detailed clinical reports documenting CHI are lacking. Interestingly, experimental studies have shown that adipocytes of SGBS patients are more sensitive to insulin stimulation, which may cause increased glucose uptake and thereby cause hypoglycemia (33).

Neonatal hypoglycemia has anecdotally been reported in **Weaver syndrome** (OMIM #277590), an overgrowth syndrome with variable intellectual disability and characteristic facial features, caused by heterozygous EZH2 pathogenic variants (34), but CHI has not been documented in the literature, so far.

**Perlman syndrome** (OMIM #267000) is an autosomal recessive condition caused by DIS3L2 mutations. Major clinical features neonatal macrosomia, facial anomalies, renal dysplasia, nephroblastomatosis and multiple congenital anomalies. It is associated with high neonatal mortality (35). Neonatal hypoglycemia was repeatedly reported. Autopsy in one patient that died unexpectedly at 8 months of age revealed an increase in the number of the pancreatic islets, leading to the speculation that hyperinsulinism might play a role for fetal macrosomia and postnatal complications (36). In fact, CHI has not been documented in literature, to date.

Foster et al. reported infantile hypoglycemia in **Kosaki overgrowth syndrome** (OMIM #616592) caused by PDGFRB mutations (37). Tenorio et al. reported neonatal hypoglycemia in some of the patients affected by RNF125-related overgrowth syndrome (**Tenorio syndrome**; OMIM #616260) (38). In both cases there was not a diagnosis of hyperinsulinism. The wide range of prenatal overgrowth syndromes with a reported association with neonatal hypoglycemia suggests that a predisposition to disturbed neonatal glucose regulation might be a feature that is common to this group of disorders in more general.

### 3.4 PI3K-AKT pathway disorders

Human disorders caused by activating mutations in the PI3K-AKT pathway constitute a large group of diseases including Proteus syndrome (OMIM #176920), PIK3CA-related overgrowth spectrum (OMIM #612918), megalencephaly-polymicrogyria-polydactyly-hydrocephalus (MPPH; OMIM #PS603387) syndrome and others. Most of these entities are sporadic with mosaicism for the causative mutation, but in a few cases germline mutations have also been observed. The activating AKT2 p.Lys17Glu mutation (mosaic or germline) was identified in children with (asymmetric) overgrowth and severe recurrent hypoglycemia from infancy with a classical biochemical profile of hyperinsulinism (i.e., low serum levels of ketone and free fatty acids), but undetectable insulin (OMIM #240900) (39). Several other similar cases have been reported (40–42). AKT2 seems to be consistently associated with this metabolic phenotype. Further publications pointed out that hypoglycemia with a similar biochemical profile may also occur in a subset of patients with other PI3K-AKT pathway disorders caused by (mosaic) activating mutations in AKT3, PIK3CA, PIK3R2, and CCND2, thus suggesting that this type of metabolic dysregulation is in principle shared by the entire group of disorders (43, 44). Consistent with this, Saito et al. described a case of AKT1-caused Proteus syndrome with hypoinsulinemic hypoglycemia (45), and Liu et al. reported a case with PTEN-related overgrowth (OMIM #158350) and recurrent hypoketotic hypoglycemia (46). Onset of hypoglycemia in patients with PI3K-AKT pathway disorders was variable, mostly within the first years of life but not typically neonatal.

It is assumed that uncoupling of cellular responses to insulin that are mediated by the PI3K-AKT pathway, such as membrane translocation of the glucose transporter GLUT4, is the underlying mechanism (39). The hypoinsulinemic hypoglycemia of PI3K-AKT pathway disorders is therefore considered a mimicker or phenocopy of CHI (5). The clinical heterogeneity and apparent low penetrance of the metabolic phenotype (except for AKT2) may be explained by the mosaicism nature of these disorders. Leiter et al. speculated that more widespread distribution of mosaicism that involves also metabolic target organs like the liver is required to produce the hypoglycemia phenotype (43). A mouse model using inducible ubiquitous knock-in of the constitutively active Pik3ca^H1047R^ mutation reproduced the human phenotype with overgrowth and metabolic abnormalities, including a reduction in blood glucose levels and undetectable insulin levels, thereby underscoring the critical role of PI3K in the regulation of glucose metabolism (47). Dushar et al. described response to sirolimus treatment in a family with familial hypoinsulinemic hypoglycemia caused by the recurrent AKT2 mutation (48).

### 3.5 Kabuki syndrome

Kabuki syndrome (OMIM #PS147920) is a developmental disorder characterized by recognizable facial features, intellectual disability, postnatal growth deficiency and variable congenital malformations. Pathogenic variants in the autosomal gene KMT2D or in the X-chromosomal gene KDM6A account for approximately 75% and 3-5% of cases, respectively (49). Both genes encode for histone-modifying enzymes, thus placing Kabuki syndrome in the group of disorders of chromatin regulation.

Neonatal hypoglycemia appears to be quite frequent in Kabuki syndrome and several cases of CHI have been reported. CHI may be the presenting feature of the disease (50). However, the prevalence figures in the literature are conflicting: In a congress report by de Leon et al. it was mentioned that in up to 70% of children with Kabuki syndrome CHI was observed (51), a prevalence figure that was cited by others, although no reference to original data has been provided. Genevieve et al. presented a series of 20 patients and reviewed 313 published cases with an overall prevalence of approximately 7% for neonatal hypoglycemia and 2% for persistent hypoglycemia and/or CHI (52). These figures are likely to be an underestimation, as it cannot be assumed that a proper assessment for neonatal hypoglycemia and CHI was conducted for each of these patients. CHI in Kabuki syndrome has also been described in a number of case reports (53–59). Yap et al. reported 10 cases of Kabuki syndrome with CHI/HH and suggested that the rate of hyperinsulinism among patients with Kabuki syndrome might be higher than previously assumed (50). In a series of 69 patients with syndromic HH, Kostopoulou et al. reported 9 cases of Kabuki syndrome (5), making it the second most common diagnosis in this cohort after BWS.

Notably, there is convincing evidence of significant genotype-phenotype correlations with a higher prevalence of neonatal hypoglycemia and hyperinsulinism in patients with KDM6A-caused Kabuki syndrome (50, 60, 61). Faundes et al. presented a large cohort of KDM6A-caused Kabuki syndrome and a review of the literature yielding a prevalence in the overall cohort of 56% for neonatal hypoglycemia and 28% for hyperinsulinism (61). This suggests that – although the predisposition to hyperinsulinism applies to Kabuki syndrome, in general – the defect of KDM6A may more specifically impact β-cell function compared to a defect of KMT2D (60). The precise mechanism for CHI in Kabuki syndrome remains to be elucidated. Considering the molecular basis of this syndrome it is conceivable that epigenetic mechanisms in metabolic regulation are affected by the underlying defect in chromatin modification.

The majority of patients with Kabuki syndrome and CHI respond to diazoxide and hyperinsulinism typically resolves within the first two years of life (5, 51). However, few patients also underwent pancreatectomy (5) (**Supplementary Table S1**).

### 3.6 Costello syndrome

Costello syndrome (OMIM #218040) belongs to the RASopathies, a group of developmental syndromes caused by mutations in components or modulators of the RAS-MAPK pathway. Costello syndrome is characterized by congenital heart defects, myocardial hypertrophy, feeding difficulties, failure to thrive in infancy, postnatal growth delay, distinctive craniofacial features, developmental delay/intellectual disability, and tumor predisposition. Specific activating HRAS variants (most commonly p.Gly12Ser), which are also known as somatic oncogenic mutations, are causative for this disease (62). The HRAS gene is located on chromosome 11p15.5 close to the BWS region (**Figure 1D**).

Neonatal hypoglycemia is quite common in Costello syndrome, it has been reported with a frequency of 44% (63). CHI, however, has only occasionally been documented (64–66). Sheffield et al. reported one case with Costello syndrome and CHI where autopsy identified a pancreatic nodule with morphologic and immunohistochemistry findings similar to a focal lesion of CHI. No K_ATP_ channel mutation was detected (65). In another patient with severe neonatal hypoglycemia, Kerr et al. reported a “nesidioblastosis-like” lesion with islet hypertrophy and hyperplasia (67). Gripp et al. demonstrated patUPD11p in the focal lesion from the patient reported by Sheffield et al. (68), thus suggesting a similar pathophysiology for CHI as in BWS in this particular case (**Figure 1D**). Notably, patUPD11p is known as a somatic driver event in Costello syndrome-associated tumors (69). It remains questionable that all cases of CHI in Costello syndrome are accounted for by this mechanism. No such investigations have been reported in other patients. Given the frequency of neonatal hypoglycemia in Costello syndrome, it seems that intrinsic mechanisms driven by mutant HRAS itself are involved, which are not known in detail. Notably, metabolic disturbances with hypoglycemia and hypercholesterolemia have been recognized as a frequent finding in Costello syndrome beyond infancy (70). Oba et al. generated mice with the Costello syndrome-associated Hras mutation G12S and observed hypoketosis and elevated levels of long-chain fatty acylcarnitines under starvation conditions suggesting impaired mitochondrial fatty acid oxidation. They concluded that the mutant Hras modulates energy homeostasis *in vivo* (71).

Data on management and long-term outcome of CHI in Costello syndrome are scanty. Responsiveness to diazoxide has been reported; one patient underwent pancreatectomy (12) (**Supplementary Table S1**).

Neonatal hypoglycemia has also been reported in other RASopathies (63), but it occurs less frequently than in Costello syndrome (9% in Noonan and 6% of cardio-facio-cutaneous syndrome patients) and cases of documented CHI are lacking.

### 3.7 Turner syndrome

Turner syndrome is a relatively common sex chromosome abnormality affecting approximately 1 in 2,500 live female births. It is caused by monosomy X (karyotype: 45,X) or various other X-chromosomal abnormalities leading to partial monosomy. Short stature and primary amenorrhea due to ovarian dysgenesis are hallmarks of the disease. Several cases of CHI/HH in girls with Turner syndrome or mosaic Turner syndrome have been reported (72–74), suggesting an increased incidence of CHI/HH in this disorder, the precise dimension of which remained however undetermined. Gibson et al. presented 12 girls with Turner syndrome in combination with CHI (75). Based on their patient cohort, the authors estimated that the risk of CHI/HH in girls with Turner syndrome might be increased by about 50-fold compared to general population (75). Kostopoulou et al. identified 6 cases of Turner syndrome in their cohort of 69 patients with syndromic CHI/HH, making Turner syndrome the third most common syndromic cause of CHI/HH in this series (5).

The underlying mechanism leading to hyperinsulinism in Turner syndrome remains unclear. It has been speculated that the loss of one copy of the KDM6A gene which is located on Xp might play a role, thus suggesting a similar mechanism for CHI as in KDM6A-related Kabuki syndrome (75). This notion was supported by the finding that islets isolated from the pancreas of one Turner syndrome patient showed abnormal regulation of insulin secretion, with increased sensitivity to amino acids and elevated basal cytosolic calcium, a phenotype that could be partially reproduced in mouse islets exposed to a KDM6A inhibitor (75).

A majority of the reported cases with Turner syndrome and CHI were responsive to diazoxide and resolution of HH frequently occurred within the first years of life (5, 75) (**Supplementary Table S1**). Few patients underwent pancreatectomy with a histopathology consistent with diffuse hyperinsulinism (75). The susceptibility to abnormal glucose homeostasis in infancy may be related with the increased susceptibility to insulin resistance and β-cell dysfunction which is a well-known feature in adolescent and adult females with Turner syndrome (76, 77).

### 3.8 Chromosome 9p deletion syndrome

Variably sized monosomy of the short arm of chromosome 9 may result from isolated deletions or unbalanced translocations. Terminal 9p deletions cause complex syndromic conditions with multiple congenital anomalies and developmental delay/intellectual disability (OMIM: #158170). CHI has been observed in a number of cases with monosomy 9p: Banerjee et al. reported 12 cases with neonatal hypoglycemia, ten of them with biochemically confirmed hyperinsulinism, and reviewed three previously reported cases (78). Kostopoulou et al. observed one case with a chromosome 9 deletion and chromosome 2 duplication (not further specified) among 69 patients with syndromic neonatal hypoglycemia, but CHI was not confirmed in that case (5). The prevalence of CHI in patients with monosomy 9p is unknown, but it has been recognized in less than 10% of cases reported in the literature and databases.

The precise molecular mechanism of the dysregulated insulin secretion associated with monosomy 9p remains unknown. A minimal deleted region was mapped to 7.2 Mb, encompassing 38 protein-coding genes. SMARCA2 and RFX3 were proposed as potential candidates for the hypoglycemia, but no experimental evidence has been provided (78).

The course of CHI in monosomy 9p may be transient or persistent; responsiveness to diazoxide with treatment up to the age of 6 has been reported (**Supplementary Table S1**).

### 3.9 Usher-CHI syndrome (homozygous 11p15-p14 deletion syndrome)

Usher-CHI syndrome (OMIM #606528) is the unique combination of two autosomal recessive syndromes caused by homozygosity for a recurrent 122 kb deletion which encompasses parts of the neighboring genes ABCC8 and USH1C, USH1C:c.(90 + 592)_ABCC8:c.(2694–528)del) (**Figure 1F**). The condition was first described by Bitner-Glindzicz et al. with the clinical features of CHI, congenital sensorineural deafness, developmental delay, enteropathy, and renal tubular dysfunction (79). Hussain et al. (80) and Al Mutair et al. (81) reported additional cases.

CHI in this condition is similar in its clinical presentation and pathophysiology to the ABCC8-deficient autosomal recessive diffuse form, while the remainder of the phenotype represents Usher syndrome type 1C and is explained by the USH1C defect. Accordingly, these patients do usually not respond to diazoxide, and most of the published cases underwent surgery (**Supplementary Table S1**).

### 3.10 Other chromosomal contiguous gene deletions reported as associated with CHI/HH

**Trisomy 13** or mosaic trisomy 13 have been anecdotally reported in association with CHI (82–84). Shiu et al. added a case of CHI with partial trisomy 13: 47,XY,+del(13)(q14q32) (85). Kostopoulou observed another case in their series of 69 children with syndromic CHI (5). The potential molecular mechanism underlying CHI in trisomy is unclear and other genetic causes of CHI have not been excluded in those children.

Kostopoulou et al. reported two patients with CHI and a **16p11.2 microdeletion** (OMIM #613440) (86). Hoytema van Konijnenburg observed CHI in a patient with Zellweger syndrome who was found to have also a 16p11.2 microdeletion. Based on the lack of descriptions of CHI in Zellweger syndrome and the previous publication by Kostopoulou et al., the authors discussed that CHI in their patient was most likely caused by the 16p11.2 deletion syndrome (87). The 16p11.2 recurrent deletion phenotype includes a variable spectrum of developmental delay/intellectual disability, psychiatric conditions, autistic features, and epilepsy (88). Notably, early-onset obesity is a known feature of this disorder (88, 89) and may be associated with secondary hyperinsulinism (90). This might point towards a possible primary metabolic alteration in this syndrome. However, it has to be taken into account the prevalence of approximately 1:2000 live births in the general population for 16p11.2 microdeletions. Therefore, a chance association for the few reported cases with CHI cannot be excluded. Baple et al. et al. reported one case of syndromic CHI with a *de novo* interstitial **12q24.31 deletion**. The authors discussed that the CHI phenotype could be accounted for by haploinsufficiency of HNF1A which was contained in the deleted interval (91).

Kostopoulou reported one case of **trisomy 21** among 69 children with syndromic CHI (5). No such cases had been described before. However, more recently Hewat et al. reviewed cases referred for genetic testing of CHI in a national reference center. They identified 11 individuals with Down syndrome in a cohort of 2011 patients referred for genetic testing for CHI, which represents an increased prevalence compared to the general population (92). A pathogenic ABCC8 mutation was identified in one of the 11 individuals, probably explaining the CHI phenotype in this child. Five others were reported to have non-genetic risk factors for hyperinsulinism resulting from co-morbidities (intrauterine growth retardation, prematurity, gastrointestinal surgery possibly leading to dumping syndrome, L-asparaginase treatment). Similar results were reported in a retrospective study from Finland where five cases with Down syndrome were identified in a cohort of 238 individuals, one of them with a pathogenic heterozygous KCNJ1 variant (93). In eight of the cases reported in these two studies CHI was reported as persistent (including the two cases with K_ATP_ mutation) and in seven as transient. The majority of children requiring medical therapy responded to diazoxide; one received surgery (**Supplementary Table S1**). Hewat et al. concluded that the overrepresentation of Down syndrome in cohorts referred for CHI testing was likely due to an increased burden of non-genetic risk factors resulting from the Down syndrome phenotype (92).

The hypothesis raised by Hewat et al. regarding trisomy 21 (92) may also apply for other chromosomal disorders that have anecdotally been associated with CHI, including trisomy 13, which is discussed above. Kostopoulou et al. also reported one case of monosomy 22q11.2 (DiGeorge syndrome), as well as eight cases with other chromosomal anomalies (duplications/deletions) in their series of 69 cases with syndromic CHI (5). Giri et al. reported a patient with CHI and Poland syndrome, who had a 10p13–14 duplication (94).

### 3.11 CDG syndromes

Congenital disorders of glycosylation (CDG; OMIM #PS212065) constitute a large heterogeneous group of rare genetic disorders of glycan synthetic pathways with mainly autosomal recessive inheritance. They have a wide phenotypic spectrum with multisystem involvement including failure to thrive, developmental delay, neurologic and ocular abnormalities, hepatopathy, enteropathy and others (95). The genes causing CDG syndromes encode enzymes of the glycan synthetic or interacting pathways. The estimated prevalence of CDG syndromes in Europe is 1:22,000 live births; the most common defect is PMM2-CDG, followed by ALG6-CDG, ALG1-CDG, and MPI-CDG (96).

Recurrent hypoglycemia is a known feature in CGD syndromes, and several cases have been described with neonatal or infantile HH. The entities reported to be associated with hyperinsulinism are PMM2-CDG (CDG1a), MPI-CDG (CDG1b), ALG3-CDG (CDG1d), and PGM1-CDG (CDG1t) (reviewed by 97, 98). This distribution seems to be not significantly different from the general prevalence of CDG subtypes, suggesting that disturbed blood glucose regulation is rather a disease group feature than specific for certain entities. However, it can be noted that HH seems to be particularly common in MPI-CDG where hypoglycemia was reported in a majority of patients with a mean age of presentation of 6.8 months and hyperinsulinism in two thirds of hypoglycemic patients (99). CHI may be the presenting sign of MPI-CDG (100, 101). Well-documented cases of hypoglycemia in CDG syndromes are otherwise rare. For the most common type, PMM2-CDG (CDG1a), Vuralli counted 37 affected children among 1060 published cases (3,4%) (98). Manifestation of hypoglycemia was mostly in the first months of life and in six of the reported cases it was the first presenting symptom. Hyperinsulinism was confirmed in about half of the cases (10/22) from which appropriate clinical and laboratory data were published, and the majority of them responded to diazoxide. The authors presented three new cases and proposed that hyperinsulinism might be more frequent in PMM2-CDG than previously reported (98). Wong et al. observed hypoglycemia in 89% of patients with PGM1-CDG (CDG1t), but hypoglycemia at any age was included and hyperinsulinism was not reported (102). Sun et al. reported on case of ALG3-CDG (CDG1d) with CHI and islet cell hyperplasia on autopsy (103). HH in CDG syndromes is mostly responsive to diazoxide. Oral mannose treatment has been reported to have a favorable effect on hypoglycemia in patients with MPI-CDG (99, 100).

The mechanism underlying hypoglycemia in CGG syndrome is unclear. A complex pathogenesis may be assumed, since these patients have multisystem involvement often with other endocrine, hepatic and other organ involvement. However, there is evidence that protein glycosylation may also be directly involved in glucose homeostasis. It has been demonstrated, for example, that SUR1 glycosylation is critical for the proper trafficking and surface expression of K_ATP_ channels (104).

Notably, a distinct promoter mutation (c.-167G>T) in the PMM2 gene, either homozygous or in trans with other PMM2 coding mutations, was identified in several unrelated individuals with a phenotype of HH and congenital polycystic kidney disease, who did not exhibit the typical clinical or diagnostic features of CDG1a (105). The diagnosis of HH was within the first year of life in 11 out of 17 children and in the newborn period in four. The authors proposed that the PMM2 promoter mutation might alter tissue-specific chromatin loop formation, with consequent organ-specific deficiency of PMM2 explaining the restricted phenotype. Soares et al. presented another case of CHI and polycystic kidneys with that particular PMM2 variant (106). Chen et al. recently reviewed this particular clinical and genetic subtype of CDG syndromes under the term PMM2-HI (107).

CHI associated with CDG syndromes has commonly been reported to respond to diazoxide, while our literature search revealed only one case (1 of 38) treated with pancreatic surgery (**Supplementary Table S1**).

### 3.12 Other monogenic syndromic conditions reported as associated with CHI/HH

Heterozygous mutations of **CACNA1C**, which is expressed in the Cav1.2 (α1C-containing) Ca2+ channels can cause a variety of disorders with cardiac arrhythmias as the leading symptom (Timothy syndrome, Brugada syndrome, Long-QT syndrome) with distinct genotype-phenotype correlations. Timothy syndrome (OMIM #601005) is a complex syndromic condition with a combination of prolonged QT interval, congenital heart defects, syndactyly, facial anomalies, and neurodevelopmental delay. Most patients share the same pathogenic variant (p.Gly406Arg), and a similar phenotype but without syndactyly is associated with similar but distinct pathogenic variants (108). Intermittent hypoglycemia has been observed in approximately 40% of patients with Timothy syndrome and was speculated to be accounted for by episodic dysfunction of Cav1.2 (109, 110). Only one published case with CACNA1C-related disease and confirmed CHI was retrieved by our literature search (111).

*De novo* heterozygous **CACNA1D** missense mutations have been described in two patients with CHI, cardiovascular anomalies and neurodevelopmental problems (112, 113). One of them had also primary hyperaldosteronism (113). CACNA1D mutations have previously been associated with primary aldosteronism, seizures, and neurologic abnormalities (PASNA; OMIM #615474), thus suggesting a disease spectrum that may include CHI with reduced penetrance. CACNA1D gene encodes one of several α1 subunits of L-type voltage-gated calcium channel. These channels are widely expressed in mammalian organs including pancreatic islets (114). Cav1.3 (α1D-containing), as well as Cav1.2 (α1C-containing) Ca2+ channels have differential modulatory effects on glucose-stimulated insulin secretion (115, 116).

**KCNQ1** is a gene located in the 11p15.5 differentially methylated region (**Figure 1A**) and encodes a potassium channel. A possible role of reduced expression of KCNQ1 in BWS-associated CHI has been discussed above. KCNQ1 causes Long-QT syndrome (OMIM #192500) and other types of cardiac arrhythmias. Torekov et al. pointed out that patients with KCNQ1-related Long-QT syndrome may exhibit hyperinsulinemia and symptomatic reactive hypoglycemia after glucose challenge (117). Experimental data on Kcnq1-mutant mice showing age−dependent transition from islet insulin hypersecretion to hyposecretion support the role of this potassium channel in insulin regulation (118). Similar findings as in KCNQ1-related Long-QT syndrome were made in patients with mutations in KCNH2, the second most common cause of Long-QT syndrome (OMIM #613688) (119). No well-documented reports on CHI with either KCNQ1 or KCNH2 mutations exist in the literature. In summary, it seems to be plausible that potassium channel mutations other than K_ATP_ may have impact on insulin regulation in pancreatic β-cells, but their relevance for CHI remains unclear.

**FOXA2-CHI**: Giri et al. identified a *de novo* heterozygous mutation in FOXA2 (c.505T>C, p.S169P) in a child with CHI and congenital hypopituitarism, craniofacial anomalies, choroidal coloboma, cardiovascular and malformations gastrointestinal abnormalities, and developmental delay (120). Additional single case reports described patients with hypopituitarism and HH, and variable other abnormalities, who carried *de novo* FOXA2 mutations (121, 122). FOXA2 point mutations and deletions have also been reported patients with syndromic hypopituitarism but without documented hyperinsulinism (reviewed by 124). Current evidence thus suggests that FOXA2 should be considered in the differential diagnosis of HH especially when pituitary deficiencies co-exist (95). FOXA2, also known as HNF3B, is conserved transcription factor that is involved in the development of endoderm-derived organs including the pancreas (123) and acts as an activator of genes that function in multiple pathways governing insulin secretion (121, 124). FOXA2 has also been proposed to act as a metabolic sensor in hypothalamic neurons (125), and its role in glucose metabolism is supported by the finding that tissue-specific deletion of Foxa2 in pancreatic β-cells in mice results in HH (126). We retrieved four cases with FOXA2 mutations and a confirmed diagnosis of CHI in the literature; two out of three receiving treatment with diazoxide were described as (partial responders (**Supplementary Table S1**).

Three males from one family with a variant in **EIF2S3** were reported with an unusual dysregulation of glucose fluctuating between diazoxide-responsive HH and postprandial hyperglycemia diagnosed in childhood, along with learning difficulties and hypopituitarism (127). EIF2S3 encoding a subunit of the eukaryotic translation initiation factor 2, eIF2γ, and hemizygous mutations have been associated with a more severe syndrome of developmental delay/intellectual disability, epilepsy, hypogonadism, microcephaly, and obesity (MEHMO syndrome, OMIM #300148). Neonatal hypoglycemia as well as early-onset diabetes have been observed in patients with MEHMO syndrome (128). EIF2S3-mutated individuals display a complex metabolic-endocrine phenotype that may initially resemble CHI (129).

**Congenital central hypoventilation syndrome** (CCHS; OMIM #209880) may be associated with episodic hypoglycemia, sometimes manifesting with hypoglycemic seizures (130). Hyperinsulinemia has been reported in several cases (131–134), but in only few of them the manifestation was within the first weeks of life (131, 133). Most of the patients had a typical polyalanine expansion mutation in the PHOX2B gene as usually found in CCHS without hyperinsulinism. CCHS is a disorder of autonomic dysfunction and it has been speculated that this might also explain the predisposition to disturbance of glucose homeostasis (130, 134). However, manifestation of CHI appears to be very rare in CCHS. When necessary, conventional pharmacological treatment was found efficient (**Supplementary Table S1**).

HH has been described in a small number of patients with **Rubinstein-Taybi syndrome** (RTS; OMIM # PS180849**)** (5, 135–138). Age at diagnosis of HH was variable; only few cases had well-documented neonatal onset justifying the diagnosis of CHI (135, 137). The prevalence of CHI/HH in RTS is not known, but seems to be quite low (less than 5%). A recent meta-analysis of EP300-mutated RTS identified hypoglycemia in three subjects with documented neonatal onset in two of them (139). A majority of cases with RTS and HH that have a reported genotype had EP300 mutations, which only account for about 10% of RSTS patients, in general. Kostopoulou et al. reported two cases of RTS without genotype information in a cohort of 69 patients with syndromic CHI (5). The EP300 and CREBBP genes encode p300 and CBP, respectively, which function as transcriptional coactivators with histone acetyltransferase activity (histone modification) and are – among various other functions – also involved in islet cell development (140).

Sekiguchi et al. reported a case of **CHARGE syndrome** (OMIM #214800) caused by a CHD7 point mutation with HH in infancy (141). Kostopoulou et al. reported another case (5). CHD7 encodes a chromodomain helicase DNA-binding protein involved in chromatin remodeling and transcriptional regulation. Given the estimated prevalence of approximately 1:10,000 live births (142), the occurrence of HH in CHARGE syndrome can currently not be distinguished from a chance association.

Imaizumi et al. reported a case of **Coffin-Siris syndrome** (OMIM # PS156200) presenting at 4 month of age with recurrent hypoglycemia attacks (143). Kostopoulou et al. reported another case with HH (5). Additional cases of hyperinsulinism in Coffin-Siris syndrome appear in the literature, but they are not congenital but rather obesity-associated (144, 145). Coffin-Siris syndrome is a heterogeneous disorder of the SWI/SNF chromatin remodeling complex. Current evidence for a significant role in syndromic CHI is limited.

A syndromic condition comprising CHI, renal tubular dysfunction (Fanconi syndrome) and transient or recurrent hepatic dysfunction (OMIM #616026) was reported in patients with a heterozygous mutation of **HNF4A**, a gene that is known to be associated with MODY1 (OMIM #125850), but initial presentation in infancy may be CHI (146–148): Flanagan 2010, Stanescu 2012, Hamilton 2014). Notably, the syndromic condition including CHI, renal and hepatic disease has exclusively been reported in patients with the same HNF4A missense variant, p.Arg63Trp (R63W, also known as R76W or R85W). More than 20 cases have been reported (**Supplementary Table S1**). All patients presented with renal Fanconi syndrome and showed transient CHI. Later development of MODY was reported in some. About half of them developed recurrent benign hepatic dysfunction (reviewed by 149).

Soden et al. reported siblings with a heterozygous pathogenic MAGEL2 variant (**Schaaf-Yang syndrome**; OMIM #615547), probably based on parental germ cell mosaicism, who presented with CHI (150). Another case was reported by Halloun et al. (151). Neonatal hypoglycemia was also reported in further cases with this condition but were mostly attributable to growth hormone deficiency or adrenal insufficiency, or the etiology of the hypoglycemia has not been determined (151).

In a consanguineous family with microcephaly, short stature, and hyperinsulinemic hypoglycemia, Gillis et al. identified homozygosity for the missense variant G206R in the **TRMT10A** gene. Manifestation of hypoglycemic seizures in three affected siblings was between age 5 and 9 (152). A homozygous mutation in this gene was previously reported in another consanguineous family with a syndrome of young onset diabetes, short stature and microcephaly with intellectual disability (153). Hyperinsulinemic hypoglycemia was observed in a 3 months-old individual with a homozygous **YARS** mutation (encoding a tRNA synthetase) (154). Other cases with this disorder displaying infantile hypoketotic hypoglycemia have been described (155). The validity of the association with CHI in these extremely rare recessive diseases remains to be confirmed by additional observations. Kostopoulou et al. also reported one case of Alagille syndrome and one with Prader-Willi syndrome in their series of 69 cases with syndromic CHI (12), but no details were provided leaving the association of CHI with these disorders uncertain.

**Tyrosinemia type 1** (OMIM #276700) is a rare metabolic disorder caused by a defect of fumarylacetoacetate hydrolase (encoded by FAH gene). It typically manifests in young infants with liver dysfunction and renal tubular dysfunction. Affected children may occasionally present with CHI (156). Sethuram et al. observed CHI also in one case of transient tyrosinemia of the newborn, which is a benign condition with a maturational defect of the enzymes associated with tyrosine metabolism without any genetic abnormalities (157). CHI in tyrosinemia type 1 is responsive to diazoxide and usually resolves within the first years of life (156, 157). Although islet-cell hypertrophy and hyperplasia have been reported in a number of cases of tyrosinemia type 1 (158), the precise pathophysiology of hyperinsulinism remains obscure. Hyperinsulinemic hypoglycemia may also be a presenting sign in other inherited metabolic diseases that may manifest with a complex syndromic phenotype, such as **adenosine kinase deficiency** (OMIM #614300) (159).

## 4 Discussion

The chromosomal region 11p15 plays a key role in CHI, as it contains the genes for the two components of the islet cell-specific K_ATP_ channel in 11p15.1. The 11p15 region is also involved in several syndromic forms of CHI (**Figure 1**), first and foremost BWS, which represents the most common form of syndromic CHI (5). However, the mechanisms of abnormal glucose regulation in 11p15-related disorders are variable and are not directly linked to defective K_ATP_ channel function in all of them (e.g. most cases with Beckwith-Wiedemann syndrome, Costello syndrome). Dysregulated expression of genes of the differentially methylated region in 11p15.5 is likely to play an important role, but their impact on metabolic programming the disturbance of which can lead to CHI is incompletely understood.

The broad and heterogeneous spectrum of syndromic disorders having reported associations with CHI/HH is intriguing. A few disease groups stand out from this diverse mixture and point at shared pathophysiologies: Overgrowth syndromes (Beckwith-Wiedemann syndrome, Sotos syndrome and others) seem to convey particular susceptibility to neonatal and infantile hypoglycemia with or without proven CHI. The abnormal metabolic and growth regulation that leads to intrauterine overgrowth may at the same time predispose to hypoglycemia and inappropriate insulin secretion in early postnatal life. Thorough investigation of patients presenting with neonatal hypoglycemia may in future reveal cases with CHI-like patterns in prenatal overgrowth conditions where CHI has not been documented, so far. This notion is supported by the observation of neonatal hypoglycemia in several rare overgrowth syndromes, such as Simpson-Golabi-Behmel, Weaver, Perlman, Kosaki and Tenorio syndromes (32, 34–38). For channelopathies that are associated with syndromic forms of CHI the shared mechanism leading to hyperinsulinism is probably their role in the regulation of ion currents in pancreatic β-cells, while additional manifestations reflect the function in other organs (e.g. the myocardium). Syndromic disorders associated with impaired glucose metabolism and early-onset diabetes may manifest with CHI in early infancy as a common pattern (HNF4A, HNF1A microdeletion, EIF2S3). Transient HI followed by impaired glucose tolerance and diabetes was also described in FOXA2-related CHI (122).

It is notable that among the disorders where CHI has been observed in a syndromic context, there are a number of variable chromosomal aneuploidies as well as several disorders of chromatin regulation. These may have in common the disturbance of the fine-tuning of gene expression, which does also play an important role in the fetal and neonatal metabolic programming. The precise mechanisms of CHI in these conditions remain obscure and are probably complex. If the hypothesis is true that a broad range of disturbances in the fine regulation of gene expression and cellular programming may affect the delicate shift in metabolic adaptation at the transit from intrauterine to postnatal life, it can be expected that cases of CHI/HH will also be occasionally observed in a variety of other syndromic disorders, in future. Such a hypothesis of metabolic maladaption would be consistent with the observation that CHI/HH was mostly transient in those disorders. On the other hand, it has to be considered that increased risk of CHI/HH in such complex disorders may also be due to an increased burden of non-genetic risk factors resulting from the underlying disease, such as intrauterine growth restriction, prematurity, and gastrointestinal or cardiac malformations (91).

It has to be pointed out that for many of the conditions discussed in this review – particularly in the group of chromosomal and monogenic developmental syndromes – CHI has only been documented in a small number of cases. Even for syndromes with a well-established association to CHI, the rate of affected individuals hardly reaches 10%. For those conditions, where only a few or single anecdotal reports exist, chance associations cannot be excluded and reporting of additional cases as well as experimental studies are necessary to further corroborate the causal link. However, it is also possible that CHI is under-diagnosed especially in disorders that present with complex medical issues, and a detailed metabolic and endocrine workup may be omitted or not reported especially in cases where the CHI features are transient, which is often the case in syndromic types. The paucity of observations of CHI in conditions for which hundreds of reported cases exist in the literature argues in favor of a complex pathophysiology in which the underlying genetic condition plays a role as predisposing factor but is alone insufficient to produce the CHI/HH phenotype.

Susceptibility to (neonatal) hypoglycemia appears to be a shared feature of the various entities within certain pathophysiologically related disease groups (e.g. RASopathies, chromatin disorders, CDG syndromes), while CHI has only been documented in distinct entities out of those groups. This suggests that the common pathogenetic mechanism within certain disease groups generally predisposes to the metabolic dysregulation but with variable severity and penetrance. In cases where neonatal hypoglycemia is only mild and transient, hyperinsulinism is likely to remain undiagnosed. In fact, many publications reporting neonatal hypoglycemia in the disorders discussed in this review, do not report detailed metabolic investigations. The metabolic dysregulation as a disease-group phenomenon with variable penetrance and expression in the different entities/genotypes also seems to apply for the PI3K-AKT pathway disorders, where a distinctive mechanism of uncoupling of cellular metabolic response from insulin leads to the specific non-hyperinsulinemic CHI phenocopy.

In most of the CHI-associated syndromic conditions, the precise mechanism of dysregulation of glucose-sensing and/or insulin secretion is not completely understood and not directly related to known genes involved in isolated CHI. As mentioned above, in many of them the association with CHI is inconsistent and the metabolic disturbance is transient. However, since neonatal hypoglycemia is an early sign of possible compromise in the newborn, which requires immediate diagnostic efforts and intervention, this symptom may be the first to bring a patient to medical attention. Untreated hypoglycemia poses individuals affected by CHI at risk for central nervous system complications (1). Thereby, the consequences of CHI can add to neurodevelopmental deficits in syndromic disorders, the contribution of which may be difficult to delineate.

As mentioned above, diagnosis of a primary disorder of glucose regulation may be delayed or even be missed in complex clinical szenarios, when an infant has multiple clinical issues and medical interventions. Observations of neonatal hypoglycemia as a recurrent feature in some of the syndromic conditions discussed in this review with scarce documented cases of CHI may be taken as an indication of possible missed diagnoses of transient hyperinsulinemia in those disorders. As a consequence, it has been recommended that recurrent hypoglycemia should be assessed thoroughly in children with a syndromic clinical presentation. And children with features suggestive of syndromes associated with CHI/HH must be closely monitored for hypoglycemia and, when detected, be screened for possible hyperinsulinism (5). Identifying the genetic cause of CHI in a newborn with associated congenital anomalies or additional medical issues remains a differential diagnostic challenge and may require a broad genetic workup (5, 12, 160, 161). Depending on the clinical presentation, this should include testing for BWS, particularly patUPD11p, microarray analysis to detect chromosomal aneuploidies, as well as the analysis of a number of genes by multi-gene panel, exome or genome sequencing. In a recent review, Hewat et al. pointed out the usefulness of a comprehensive screening using targeted gene panels, exome, or genome sequencing for genetic testing for CHI, but it should be recognized that limitations remain with next-generation sequencing, and additional investigations (e.g. for the detection of copy number changes and methylation defects) may be required (162). It should also be noted that the epigenetic or genomic causes for BWS, especially patUPD11p, may not be detectable in leukocyte DNA and may require other DNA sources for detection (buccal cells, fibroblasts) (6).

Identification of the underlying cause of a syndromic disease with CHI may also have impact on individual surveillance and personalized treatment. A diagnosis of BWS should lead to the recommended tumor surveillance (6). Knowing the underlying genetic condition may also help to better assess the prospects of success of diazoxide treatment or pancreatic surgery. BWS due to patUPD11p, mosaic genome-wide patUPD and Usher-CHI syndrome have the highest rates of non-responsiveness to diazoxide (50% or more) and required pancreatectomies in one third or more of the reported cases. In contrast, the other syndromic types of CHI were mostly described to respond to conventional treatment with diazoxide and/or octreotide and surgery was performed only occasionally (**Table 1** and **Supplementary Table S1**). Response to Ca2+ channel blockers has been reported for CHI caused by mutated CACNA1D (109) and a favorable effect of oral mannose treatment on HH has been observed in MPI-CDG (97, 98). More personalized therapies for rare diseases are likely to emerge in the future.

Finally, genetic counseling should generally be offered to parents of a child diagnosed with a syndromic disorder. Many of the diseases reviewed here are due to *de novo* dominant mutations and have a low risk of recurrence in the affected family (**Table 1**). However, the rare possibility of parental germ cell mosaicism cannot be excluded, and recurrence of CHI in siblings has, for example, been reported in Schaaf-Yang syndrome (150). Autosomal recessive or X-linked inheritance (**Table 1**), as well as familial balanced chromosomal translocations as reported, for example, in one family with 9p monosomy (78) may be associated with a substantial risk of recurrence. Since several of those syndromes have serious consequences on health and life quality besides the ones conferred by CHI itself, prenatal counseling and genetic testing may be indicated.

## 5 Conclusions

Syndromic disorders that have been found to be associated with CHI/HH comprise a very heterogeneous spectrum of diseases. For several of them the association is only supported by a few observations, but undiagnosed cases are likely to exist particularly in conditions where CHI is only transient. The pathophysiology underlying CHI remains obscure for many of these disorders, and the wide spectrum of syndromes with very different genetic causes suggest that the list of syndromes with occasional manifestation of CHI will further increase. A broad genetic workup is recommended for newborns or infants presenting with CHI/HH and associated congenital anomalies or additional medical issues.

## Author contributions

MZ contributed to the conception and the writing of the article. KP gave the constructive discussions to the article. KM revised important intellectual content critically for important intellectual content. All authors contributed to the article and approved the submitted version.
